# Heterosubtypic Antibody Response Elicited with Seasonal Influenza Vaccine Correlates Partial Protection against Highly Pathogenic H5N1 Virus

**DOI:** 10.1371/journal.pone.0017821

**Published:** 2011-03-25

**Authors:** Heng Ding, Cheguo Tsai, Fan Zhou, Philippe Buchy, Vincent Deubel, Paul Zhou

**Affiliations:** 1 Unit of Anti-Viral Immunity and Genetic Therapy, Key Laboratory of Molecular Virology and Immunology, Institut Pasteur of Shanghai, Chinese Academy of Sciences, Shanghai, China; 2 Institut Pasteur in Cambodia, Phnom Penh, Cambodia; Statens Serum Institute, Denmark

## Abstract

**Background:**

The spread of highly pathogenic avian influenza (HPAI) H5N1 virus in human remains a global health concern. Heterosubtypic antibody response between seasonal influenza vaccine and potential pandemic influenza virus has important implications for public health. Previous studies by Corti *et al.* and by Gioia *et al.* demonstrate that heterosubtypic neutralizing antibodies against the highly pathogenic H5N1 virus can be elicited with a seasonal influenza vaccine in humans. However, whether such response offers immune protection against highly pathogenic H5N1 virus remained to be determined.

**Methodology/Principal Findings:**

In this study, using a sensitive influenza HA (hemagglutinin) and NA (neuraminidase) pseudotype-based neutralization (PN) assay we first confirmed that low levels of heterosubtypic neutralizing antibody response against H5N1 virus were indeed elicited with seasonal influenza vaccine in humans. We then immunized mice with the seasonal influenza vaccine and challenged them with lethal doses of highly pathogenic H5N1 virus. As controls, we immunized mice with homosubtypic H5N1 virus like particles (VLP) or PBS and challenged them with the same H5N1 virus. Here we show that low levels of heterosubtypic neutralizing antibody response were elicited with seasonal influenza vaccine in mice, which were significantly higher than those in PBS control. Among them 2 out of 27 whose immune sera exhibited similar levels of neutralizing antibody response as VLP controls actually survived from highly pathogenic H5N1 virus challenge.

**Conclusions/Significance:**

Therefore, we conclude that low levels of heterosubtypic neutralizing antibody response are indeed elicited with seasonal influenza vaccine in humans and mice and at certain levels such response offers immune protection against severity of H5N1 virus infection.

## Introduction

Influenza A viruses are segmented, negative-strand RNA viruses in the family *Orthomyxoviridae*. On the basis of the antigenicity of HA and NA glycoproteins, influenza A viruses are classified into 16 HA subtypes (H1–H16) and 9 NA subtypes (N1–N9). To date, human influenza A virus subtypes have been limited to H1, H2 and H3 and to N1 and N2 [1]. Other subtypes, such as H5, H7, H9 and N7, that have been isolated from avian hosts, cause sporadic human outbreaks [2], [3].

Neutralizing antibody responses play an important role in prevention and clearance of influenza A virus infection. However, neutralizing antibodies are completely protective against secondary infections only with closely related influenza A strains, but ineffective against viruses with major antigenic divergence. Because of this, current seasonal influenza vaccines, which consists of human influenza A viruses of H1N1 and H3N2 subtypes and an influenza B virus, are prepared annually on the basis of WHO forecasts on the most probable influenza A and B virus strains thought to be circulating in the next seasonal outbreak [4], [5]. From the public health point of view, it is important to determine if antibody responses elicited with seasonal influenza vaccines can cross neutralize other HA subtypes that caused outbreaks in humans.

Previously, Gioia *et al* reported that influenza vaccination boosted heterosubtypic immunity against H5N1 viruses and the immunity involved both cytotoxic T cell and antibody responses. A rise of neutralization titer against H5N1 viruses after the seasonal influenza vaccination was only detected by microneutralization (MN) assay, but not hemagglutinin inhibition (HI) assay [6]. However, in a small pilot study using similar immunogens and immunization protocol for the same influenza season, Tang *et al* found no heterosubtypic antibody responses against H5N1 viruses from seasonal influenza vaccine in human measured by both MN and HI assays [7]. More recently, Corti *et al.* investigated the human heterosubtypic antibody response following seasonal influenza vaccination and reported that serum IgG antibodies in some vaccinated individuals cross-react with H5 HA. Furthermore, by immortalizing memory B cells from those individuals, a panel of 20 heterosubtypic neutralizing monoclonal antibodies that bound and neutralized viruses belonging to H1, H2, H5, H6 and H9 subtypes were isolated [8]. Thus, heterosubtypic neutralizing antibodies against highly pathogenic H5N1 virus and other HA subtypes could indeed be elicited with seasonal influenza vaccine in humans. However, it remains to be determined whether such heterosubtypic neutralizing antibody responses offer immune protection and if so, what neutralizing antibody titers are required for immune protection.

Previously, we developed a sensitive influenza HA and NA pseudotype-based neutralization (PN) assay [9]. We showed that although there is an excellent correlation in neutralizing antibody titers measured by the HI, MN and PN assays, in serum samples with low neutralizing antibody activity the PN assay exhibits greater sensitivity than the HI and MN assays.

Thus, in this study, using the sensitive PN assay we first tested if antibody responses elicited with seasonal influenza vaccines in healthy individuals can cross-neutralize H5N1 viruses. We hypothesized that heterosubtypic neutralizing antibody response against H5N1 viruses elicited with seasonal influenza vaccines may be too low to be consistently detected by conventional HI and MN assays, but can be readily detected by the sensitive PN assay. To accomplish this, 12 healthy volunteers were recruited and vaccinated with 2008–2009 seasonal influenza vaccines. Neutralizing antibody responses in the pre- and post-immune serum samples were evaluated by two modified PN assays - PN entry assay and PN release/entry assay - against a panel of HA and NA pseudotypes derived from H1N1, H3N2 and H5N1 viruses. We then immunized mice with the seasonal influenza vaccine, homosubtypic H5N1 VLP or PBS control and challenged them with lethal doses of highly pathogenic H5N1 virus. Neutralizing antibody responses against H1N1, H3N2 and H5N1 viruses in pre- and post-immune sera were evaluated using PN entry assay. Here we show that low levels of heterosubtypic neutralizing antibody response were indeed elicited with seasonal influenza vaccine in humans and in mice. Among them 2 out of 27 mice whose immune sera exhibited similar levels of neutralizing antibody response as VLP controls survived from highly pathogenic H5N1 virus challenge. Therefore, we conclude that low levels of heterosubtypic neutralizing antibody response can indeed be elicited with seasonal influenza vaccine in humans and mice and such response, when reached at certain levels, offers immune protection against severity of H5N1 virus infection.

### Ethical Statement

Our human study was approved by the research and ethics committee in the Institut Pasteur of Shanghai. All human participants gave written consent. Our animal study was approved by animal research committee in Pasteur Institute in Cambodia and was carried out according to international guideline of animal research.

## Materials and Methods

### Study Population

Twelve healthy young adult volunteers who were going to receiving 2008–2009 seasonal influenza vaccine gave written consent for sera to be taken prior to and after the vaccination at the Institut Pasteur of Shanghai, Chinese Academy of Sciences. The study was approved by the Institution Ethic Committee. The vaccine formulation was Vaxigrip, an inactivated split influenza vaccine (Sanofi Pasteur, Lyon, France). The antigen composition and strains were A/Brisbane/59/2007 (H1N1), A/Brisbane/10/2007 (H3N2) and B/Florida/4/2006 like. Each 0.5 ml vaccine dose contains 15 µg HA of each strain in PBS and excipients. Vaccine was administered intramuscularly. Blood samples were drawn 1 or 2 months before and 10 days after the vaccination. After clotting at room temperature for 6 hours and then at 4°C overnight, serum samples were collected, heat-inactivated at 56°C for 30 minutes and stored in aliquot at −80°C.

### Cell lines

The packaging cell line 293T [9] was maintained in complete DMEM medium [i.e. high glucose DMEM supplemented with 10% FBS, 2 mM L-glutamine, 1 mM sodium pyruvate, penicillin (100 U/ml), and streptomycin (100 µg/ml); Invitrogen Life Technologies] containing 0.5 mg/ml of G418. Madin-Darby canine kidney (MDCK) cell lines [9] were maintained in complete DMEM medium.

### Viruses

HPAI H5N1 viruses A/Shenzhen/406H/06 and A/Cambodia/P0322095/05 were originally isolated from H5N1 infected human patients at the Donghu Hospital in Shenzhen, China [10] and the Pasteur Institute of Cambodia [11], respectively. Human influenza A H1N1 virus A/WSN/33 was provided by Dr. Toyoda in the Institute Pasteur of Shanghai. Viruses were propagated in MDCK cells. Virus stocks were collected and stored in aliquot at −80°C. The fifty percentage tissue culture infection dose (TCID_50_) of virus stocks was determined by infecting MDCK cells with serial 10-fold dilution of virus and calculated by the method of Reed and Muench [12]. To determine 50% mouse lethal dose (MLD_50_) of the HPAI H5N1 virus A/Shenzhen/406H/06, groups of 5 mice were inoculated i. n. with serial 10-fold dilution of virus. After the inoculation, mice were monitored daily for 14 days. Any mouse that lost more than 25% of its initial body weight was euthanatized. MLD_50_ was calculated by the method of Reed and Muench [12]. All research with HPAI H5N1 viruses was conducted in bio-safety level 3 (BSL-3) facilities at the Pasteur Institute of Cambodia under the guidance of the Institutional Animal Care and Use Committee.

### HA and NA pseudotype-based neutralization (PN) assay

HA and NA pseudotype panel used in this study was generated as described previously [9]. The PN assay was carried out also as described before [9] with some modifications.

### 1. PN entry assay

To test effect of pre- and post-immune sera on the entry of HA and NA pseudotypes, MDCK cells (2×10^4^ cells per well) were seeded onto 24 well culture plate in complete DMEM overnight. Serially 4-fold diluted human sera or serially 2-fold diluted mouse sera (starting at 1∶10 dilution) were then incubated with various pseudotypes equivalent to 100,000–500,000 relative luciferase activity (RLA) at the final volume of 50 µl at 37°C for 1 hour. The mixture was added onto MDCK cells and incubated for 2 days. RLA in the cell lysates was measured as described before [9]. The % inhibition was calculated by (RLA in pseudotypes and medium control – RLA in pseudotypes and immune serum in a given dilution)/RLA in pseudotypes and medium control. IC50 and IC90 were determined as the dilutions of a given serum sample that result in 50% and 90% reduction of luciferase activity, respectively.

### 2. PN release/entry assay

To test the effect of pre- and post-immune sera on HA and NA pseudotype release, 4×10^5^ 293 T packaging cells were co-transfected with 1.4 µg transfer vector pHR'CMV-Luc [13], 1.4 µg packaging vector pCMVΔR8.2 [14], 0.2 µg CMV/R-HA and 0.05 µg CMV/R-NA or with 1.4 µg pHR'CMV-Luc, 1.4 µg pCMVΔR8.2 and 0.5 µg CMVR-VSV-G control in 12 well tissue culture plate as described before [9]. After overnight incubation, cells were cultured in complete DMEM supplemented with 100 µM sodium butyrate for 8 hrs. Cells were then cultured in 1 ml of complete DMEM overnight in the presence of serially 4-fold diluted sera (starting at 1∶10 dilution). Culture supernatants were collected and used to transduce MDCK cells overnight as described before [9]. Forty eight hours after the transduction, transduced cells were harvested and the luciferase activity was measured as described above.

### Micro-neutralization (MN) assay

MDCK cells (1.5×10^4^ cells per well) were seeded onto 48 well culture plate in complete DMEM overnight. To test neutralization activity of human pre- and post-immune serum samples, serially 4-fold diluted sera (starting at 1∶10 dilution) were incubated with 100 TCID_50_ wild type viruses A/Shenzhen/406H/06, A/Cambodia/P0322095/05 and A/WSN/33 at the final volume of 50 µl at 37°C for 1 hour. After the incubation, the mixture was added onto MDCK cells. The cytopathic effect (CPE) was scored at 4 days after infection as described before [9]. The assay was performed in triplicate.

### Animals

All animal protocols were approved by the Institutional Animal Care and Use Committee at the Institut Pasteur in Cambodia (Permit number VD100820). Female BALB/c mice (Mus musculus) at the age of 6 to 8 weeks were purchased from Charles River Laboratories (L'Arbresle, France) and housed in micro-isolator cages ventilated under negative pressure with HEPA-filtered air and a 12/12-hour light/dark cycle.

### Production and characterization of VLP

The production and characterization of influenza HA and NA VLP were the same as described before [9]. Briefly, 4.5×10^6^ 293T cells were co-transfected with 14 µg pCMVΔR8.2, 2 µg CMV/R-H5HA (A/Shenzhen/406H/06) and 0.5 µg CMV/R-M2 epitope-tagged N1NA (A/Thailand/1(KAN-1)/04) using a calcium phosphate precipitation method. After overnight incubation, cells were cultured in 10 ml of complete DMEM supplemented with 100 µM sodium butyrate for 8 hrs. Cells were then cultured in 10 ml of complete DMEM. The VLP-containing supernatants were harvested in 16 to 20 hrs, loaded onto 20% sucrose cushion and ultra-centrifuged at 20,000 rpm for 2.5 hours at 4°C in a Beckman SW28 rotor (Beckman Coulter, Fullerton, CA). The pellets were resuspended in PBS and stored in aliquot at −80°C.

To characterize VLP, resuspended pellets were fractionated through a 25–65% sucrose density gradient at 25,000 rpm for 16 hours at 4°C in a Beckman SW41 swing rotor (Beckman Coulter, Fullerton, CA). Twelve fractions (0.96 ml each) were collected from the top to the bottom of the gradient, TCA precipitated, separated by 12% SDS-PAGE and transferred onto PDVF membranes. Blots were blocked in a solution of Tris-buffered saline containing 5% nonfat dry milk and 0.1% Tween 20 and subsequently probed with a monoclonal antibody (clone 183-H12) specific for HIV-1 gag p24 (provided by the AIDS Reagents and Depositary program, NIAID, NIH), with a monoclonal antibody (Catalog# F3165, Sigma) specific for M2 epitope and with immune sera elicited with DNA plasmids expressing H5HA A/Shenzhen/406H/06) (see below). Antigens were visualized with an AP-conjugated anti-mouse IgG antibody according to manufacturer's instruction (Promega).

### Immunization and challenge

Two immunization and challenge experiments were performed. In the first experiment, female BALB/c mice at the age of 6 to 8 weeks were randomly divided into 3 groups (5 mice per group). Mice in group one were injected intramuscularly (i.m.) with total 200 µl PBS (pH 7.4) in both prime and boost. Mice in group two were injected i.m. with 0.5 µg (based on HA content) HA and NA VLP in total 200 µl PBS for both prime and boost. Mice in group three were injected i.m. with the same 2008–2009 seasonal influenza vaccine as used in humans (see above). Each 200 µl vaccine dose contains total 1 µg HA in PBS and excipients. The prime and the boost were carried 21 days apart. Seven days before the prime and 7 days after the boost serum samples were collected, heat-inactivated at 56°C and stored in aliquot at −80°C. Two weeks after the boost, mice in each group were challenged i.n. with 10 MLD_50_ of H5N1 virus (A/Shenzhen/406H/06, subclade 2.3.4) in a volume of 50 µl. Mice were monitored and recorded daily for 14 days post challenge. When mice lose 25% or more of their initial body weight, they were sacrificed and counted as dead mice. Virus challenge studies were conducted in BSL-3 facility at the Institut Pasteur in Cambodia in accordance with the Department of Agriculture guidelines for the Care and Use of Laboratory Animals, the Animal Welfare Act and Department of Agriculture Biosafety guidelines in Microbiological and Biomedical Laboratory.

Since in the first immunization and challenge experiment all 5 PBS control mice died, all 5 VLP control mice survived and one of 5 seasonal influenza vaccinated mice whose immune serum reached similar level as VLP controls actually survived from lethal H5N1 challenge, we carried out the second immunization and challenge experiment. In the second experiment 2 mice were injected with PBS, 2 mice with VLP and 22 mice with seasonal influenza vaccine followed by the same H5N1 challenge.

### Statistical Analysis

Each individual animal immune response was counted as an individual value for statistical analysis. The neutralizing activity was first analyzed by the Kolmogorov-Smirnov and Shapiro-Wilk normality tests for normality and then the significance was calculated by Student's *t* test. The log-rank test was used to compare the survival curves.

## Results

### Neutralizing antibody titers in pre- and post-immune sera measured by PN entry and MN assays

Tables 1 and 2 show neutralization titers (IC50) measured by PN entry assay against a panel of H1N1, H3N2 and H5N1 pseudotypes as well as VSV-G controls in pre- and post-human sera of 12 donors vaccinated with 2008–2009 trivalent seasonal flu vaccine. All H1N1 and H5N1 pseudotypes express a common N1NA. Six H5N1 pseudotypes express H5HA from clade 0 (A/Hong Kong/156/97), clade 1 (A/Vietnam/1203/04 and A/Cambodia/P0322095/05), subclade 2.1 (A/Indonesia/5/05), subclade 2.3 (A/Shenzhen/406H/06) and clade 3 (A/silky chicken/Hong Kong/SF189/01). Except for the clade 3, all other 5 H5HA were derived from human H5N1 isolates.

**Table 1:** Neutralizing antibody titers against H1N1 and H3N2 in pre- and post-immune sera measured by PN (entry) assay.

		H1N1		Vaccine antigen strain				
		A/WSN/33		H1N1 A/Brisbane/59/2007		H3N2 A/Brisbane/10/2007		VSV
	Human projects	IC50	IC90	IC50	IC90	IC50	IC90	IC50
Pre-	1	<1∶10*	<1∶10	<1∶10	<1∶10	<1∶10	<1∶10	<1∶10
immune	2	1∶10–1∶40	<1∶10	<1∶10	<1∶10	<1∶10	<1∶10	<1∶10
sera	3	<1∶10	<1∶10	<1∶10	<1∶10	<1∶10	<1∶10	<1∶10
	4	1∶40–1∶160	1∶10–1∶40	1∶10–1∶40	<1∶10	1∶10–1∶40	<1∶10	<1∶10
	5	1∶160–1∶640	1∶10–1∶40	1∶40	<1∶10	<1∶10	<1∶10	<1∶10
	6	1∶160–1∶640	1∶40	1∶10	<1∶10	1∶10	<1∶10	<1∶10
	7	<1∶10	<1∶10	<1∶10	<1∶10	<1∶10	<1∶10	<1∶10
	8	<1∶10	<1∶10	<1∶10	<1∶10	<1∶10	<1∶10	<1∶10
	9	<1∶10	<1∶10	<1∶10	<1∶10	<1∶10	<1∶10	<1∶10
	10	1∶160–1∶640	1∶40	1∶10	<1∶10	1∶10	<1∶10	<1∶10
	11	1∶40–1∶160	<1∶10	<1∶10	<1∶10	<1∶10	<1∶10	<1∶10
	12	1∶160–1∶640	1∶40	1∶40	<1∶10	<1∶10	<1∶10	<1∶10
Post-	1	>1∶2560**	1∶640–1∶2560	>1∶2560	1∶640–1∶2560	1∶640–1∶2560	1∶40	<1∶10
Immune	2	1∶640	1∶40	1∶640–1∶2560	1∶40–1∶160	1∶160–1∶640	1∶10–1∶40	<1∶10
sera	3	1∶640	1∶40–1∶160	1∶640–1∶2560	1∶40–1∶160	1∶160–1∶640	1∶10–1∶40	<1∶10
	4	>1∶2560	1∶40–1∶160	>1∶2560	1∶160–1∶640	1∶160–1∶640	1∶40	<1∶10
	5	1∶640–1∶2560	1∶160–1∶640	1∶640–1∶2560	1∶160–1∶640	1∶40–1∶160	1∶10	<1∶10
	6	1∶640–1∶2560	1∶40–1∶160	1∶640–1∶2560	1∶160–1∶640	1∶40–1∶160	1∶10	<1∶10
	7	>1∶2560	1∶640–1∶2560	>1∶2560	1∶640–1∶2560	1∶160–1∶640	1∶10–1∶40	<1∶10
	8	1∶640	1∶40	1∶640–1∶2560	1∶40–1∶160	1∶40–1∶160	<1∶10	<1∶10
	9	1∶640–1∶2560	1∶160–1∶640	>1∶2560	1∶160–1∶640	1∶40–1∶160	1∶10–1∶40	<1∶10
	10	1∶640–1∶2560	1∶160–1∶640	>1∶2560	1∶160–1∶640	1∶40–1∶160	1∶10	<1∶10
	11	>1∶2560	1∶640–1∶2560	>1∶2560	1∶640–1∶2560	1∶160–1∶640	1∶40	<1∶10
	12	1∶640–1∶2560	1∶160–1∶640	1∶640–1∶2560	1∶160–1∶640	1∶40–1∶160	1∶10–1∶40	<1∶10

*<1∶10: not detected.

**>1∶2560: the Ab titers greater than the highest antibody dilutions.

**Table 2:** Neutralizing antibody titers against H5N1 in pre- and post-immune sera measured by PN (entry) assay.

		H5N1					
		A/Hongkong/156/97 clade 0	A/Vietnam/1203/04 clade 1	A/Cambodia/P0322095/05 clade 1	A/Indonesia/5/05 clade 2.1	A/Shenzhen/406H/06 clade 2.3.4	A/Silky Chicken/Hongkong/SF189/01 clade 3
	Human projects	IC50	IC50	IC50	IC50	IC50	IC50
Pre-	1	<1∶10*	<1∶10	<1∶10	<1∶10	<1∶10	<1∶10
immune	2	<1∶10	<1∶10	<1∶10	<1∶10	<1∶10	<1∶10
sera	3	<1∶10	<1∶10	<1∶10	<1∶10	<1∶10	<1∶10
	4	<1∶10	<1∶10	<1∶10	<1∶10	<1∶10	<1∶10
	5	<1∶10	<1∶10	<1∶10	<1∶10	<1∶10	<1∶10
	6	<1∶10	<1∶10	<1∶10	<1∶10	<1∶10	<1∶10
	7	<1∶10	<1∶10	<1∶10	<1∶10	<1∶10	<1∶10
	8	<1∶10	<1∶10	<1∶10	<1∶10	<1∶10	<1∶10
	9	<1∶10	<1∶10	<1∶10	<1∶10	<1∶10	<1∶10
	10	<1∶10	<1∶10	<1∶10	<1∶10	<1∶10	<1∶10
	11	<1∶10	<1∶10	<1∶10	<1∶10	<1∶10	<1∶10
	12	<1∶10	1∶10–1∶40	<1∶10	<1∶10	1∶10	<1∶10
Post-	1	1∶40	1∶160	1∶640	1∶640	1∶160–1∶640	1∶10
immune	2	1∶40–1∶160	1∶40	1∶160	1∶160	1∶40	1∶40
sera	3	1∶10	<1∶10	<1∶10	1∶40	1∶10	<1∶10
	4	1∶160–1∶640	1∶160	1∶40	1∶160	1∶160	1∶10–1∶40
	5	<1∶10	1∶160	1∶40–1∶160	1∶10–1∶40	<1∶10	1∶160
	6	1∶40	1∶160	<1∶10	1∶160	<1∶10	1∶40–1∶160
	7	1∶160	1∶10–1∶40	1∶160	1∶40	<1∶10	1∶10
	8	1∶40	<1∶10	<1∶10	<1∶10	<1∶10	<1∶10
	9	<1∶10	<1∶10	<1∶10	<1∶10	<1∶10	<1∶10
	10	<1∶10	<1∶10	<1∶10	<1∶10	1∶40	<1∶10
	11	1∶10–1∶40	1∶40	<1∶10	1∶10	1∶10	<1∶10
	12	1∶10–1∶40	1∶40–1∶160	<1∶10	<1∶10	1∶40	<1∶10

*<1∶10: not detected.

Before 2008–2009 seasonal influenza vaccination, sera from 7, 5 or 3 of 12 donors exhibited low, but measurable neutralization titers (IC50 ranging from 1∶10 to 1∶320) against H1N1 A/WSN/33 and A/Brisbane/59/2007 or H3N2 A/Brisbane/10/2007 pseudotypes (Table 1), but none of them against H5N1 pseudotypes derived from A/Hong Kong/156/97 (clade 0), A/Cambodia/P0322095/05 (clade 1), A/Indonesia/5/05 (subclade 2.1) and A/silky chicken/Hong Kong/SF189/01 (clade 3) as well as against pseudotype expressing VSV-G control (Table 2). Sera from 1 (Subject #12) of 12 donors exhibited very low neutralization titers (IC50 from 1∶10 to 1∶40) against H5N1 pseudotypes derived from A/Vietnam/1203/04 (clade 1) and A/Shenzhen/406H/06 (subclade 2.3) (Table 2). At this moment, it is not clear whether pre-existing very low anti-H5N1 neutralizing antibody response detected in this individual is due to previous exposure to H5N1 or other influenza A viruses.

After 2008–2009 seasonal influenza vaccination, sera from all 12 donors exhibited much higher neutralization titers (IC50 ranging from 1∶640 to >2560 and IC90 ranging from 1∶40 to 1∶2,560) against H1N1 A/WSN/33 and A/Brisbane/59/2007 or H3N2 A/Brisbane/10/2007 pseudotypes, but none of them against pseudotype expressing VSV-G controls. Interestingly, post-immune sera from 11 of 12 donors also exhibited neutralization activity against at least one of H5N1 pseudotypes. Among them post-immune sera from 3 donors (Subject #1, #2 and #4) have low neutralization titers (IC50 ranging from 1∶10 to 1∶160) against all 6 H5N1 pseudotypes. Compared to the pre-immune sera, neutralization titers in post-immune sera increased at least 4 folds or higher (Tables 1 and 2).

For the comparison, neutralization titers were also measured by a standard MN assay against two representative H5N1 strains (A/Cambodia/P0322095/05 and A/Shenzhen/406H/06) and H1N1 strain (A/WSN/33). Before 2008–2009 seasonal flu vaccination, sera from all 12 donors exhibited no detectable levels of neutralization titers against all three strains; while after 2008–2009 seasonal flu vaccination, sera from 8 of 12 donors exhibited neutralization activity at the serum dilution from 1∶10 to 1∶160 against H1N1 strain; but still none of them have measurable neutralization activity against 2 H5N1 strains (Table 3), even though neutralization activity against H5N1 pseudotypes expressing H5HA isolated from the same two H5N1 strains was detected by PN entry assay (Table 2). Thus, taken together the data clearly show that in most human individuals 2008–2009 seasonal influenza vaccination elicited low, but measurable, levels of heterosubtypic neutralizing antibody responses against H5N1 viruses. However, such heterosubtypic neutralizing antibody responses can be only detected by sensitive PN entry assay, but not by MN assay.

**Table 3:** Neutralizing antibody titers in pre- and post-immune sera measured by MN assay.

		H1N1	H5N1	
	Human subjects	A/WSN/33	A/Cambodia/P0322095/05	A/Shenzhen/406H/06
Pre-	1	<1∶10*	<1∶10	<1∶10
immune	2	<1∶10	<1∶10	<1∶10
sera	3	<1∶10	<1∶10	<1∶10
	4	<1∶10	<1∶10	<1∶10
	5	<1∶10	<1∶10	<1∶10
	6	<1∶10	<1∶10	<1∶10
	7	<1∶10	<1∶10	<1∶10
	8	<1∶10	<1∶10	<1∶10
	9	<1∶10	<1∶10	<1∶10
	10	<1∶10	<1∶10	<1∶10
	11	<1∶10	<1∶10	<1∶10
	12	<1∶10	<1∶10	<1∶10
Post-	1	1∶40	<1∶10	<1∶10
immune	2	<1∶10	<1∶10	<1∶10
sera	3	<1∶10	<1∶10	<1∶10
	4	<1∶10	<1∶10	<1∶10
	5	1∶10	<1∶10	<1∶10
	6	1∶10	<1∶10	<1∶10
	7	1∶40	<1∶10	<1∶10
	8	<1∶10	<1∶10	<1∶10
	9	1∶10	<1∶10	<1∶10
	10	1∶10	<1∶10	<1∶10
	11	1∶160	<1∶10	<1∶10
	12	1∶10	<1∶10	<1∶10

*<1∶10: not detected.

### Effect of pre- and post-immune sera on release of HA and NA pseudotypes

We next performed PN release/entry assay to determine the effect of pre- and post-human immune sera on pseudotype release. To accomplish this, 293T cells were first co-transfected with pHR'CMV-Luc, pCMVΔR8.2, one of two CMV/R-H5HA (A/Cambodia/P0322095/05 and A/Shenzhen/406H/06) and CMVR-N1NA or with pHR'CMV-Luc, pCMVΔR8.2, and CMVR-VSV-G control as described before [9]. After the overnight incubation, cells were thoroughly washed and cultured in complete DMEM medium supplemented with sodium butyrate for 8 hours. Cells were then washed again and incubated in the fresh complete DMEM medium in the presence or absence of serially 4-fold diluted human pre- and post-immune sera for 18 hours. The supernatants were harvested and used to transduce MDCK cells. After the transduction, relative luciferase activity was measured. Table 4 shows IC50 of sera of 12 donors before and after 2008–2009 seasonal influenza vaccination on H5N1 pseudotype and pseudotype expressing VSV-G control measured by PN release/entry assay.

**Table 4:** Neutralizing antibody titers in pre- and post-immune sera measured by PN (release/entry) assay.

			H5N1	
	Human subjects	VSV	A/Cambodia/P0322095/05	A/Shenzhen/406H/06
Pre-	1	<1∶10*	1∶40	1∶40
immune	2	<1∶10	1∶40–1∶160	1∶10
sera	3	<1∶10	1∶40–1∶160	1∶40–1∶160
	4	<1∶10	1∶40–1∶160	1∶10–1∶40
	5	<1∶10	<1∶10	1∶10–1∶40
	6	<1∶10	<1∶10	1∶40
	7	<1∶10	1∶40	<1∶10
	8	<1∶10	<1∶10	<1∶10
	9	<1∶10	1∶10	1∶10
	10	<1∶10	1∶160–1∶640	1∶160
	11	<1∶10	<1∶10	<1∶10
	12	<1∶10	<1∶10	1∶10–1∶40
Post-	1	<1∶10	>1∶640**	1∶640
immune	2	<1∶10	1∶160–1∶640	1∶40
sera	3	<1∶10	>1∶640	1∶160–1∶640
	4	<1∶10	1∶640	1∶160–1∶640
	5	<1∶10	1∶160–1∶640	1∶40–1∶160
	6	<1∶10	1∶640	1∶40–1∶160
	7	<1∶10	1∶640	1∶160–1∶640
	8	<1∶10	1∶10	1∶160
	9	<1∶10	1∶40–1∶160	1∶40
	10	<1∶10	>1∶640	1∶160
	11	<1∶10	1∶10–1∶40	1∶40–1∶160
	12	<1∶10	1∶40–1∶160	1∶40–1∶160

*<1∶10: not detected.

**>1∶640: the Ab titers greater than the highest antibody dilutions.

Before 2008–2009 seasonal influenza vaccination, sera from 7 of 12 donors exhibited low, but measurable neutralization titers (IC50 ranging from 1∶40 to 1∶640 serum dilution) against H5N1 pseudotype A/Cambodia/P0322095/05 and sera from 9 of 12 donors exhibited low, but measurable neutralization titers (IC50 ranging from 1∶10 to 1∶160 serum dilution) against H5N1 pseudotype A/Shenzhen/406H/06, while none of them exhibited detectable levels of neutralization activity against VSV-G control. After 2008–2009 seasonal influenza vaccination, sera from 11 of 12 donors exhibited at least 4 fold increased neutralization titers against H5N1 pseudotypes A/Cambodia/P0322095/05 and A/Shenzhen/406H/06. But still none of them exhibited detectable levels of neutralization activity against VSV-G control. Sera from remaining 1 donor (Subject #10) that did not exhibit increased neutralization titers after 2008–2009 seasonal influenza vaccination actually had the highest neutralization titers in the pre-immune serum samples. Thus, these data indicate that low, but measurable levels of neutralization activity against H5N1 virus release exists even before seasonal influenza vaccination. However, after seasonal influenza vaccination, neutralization activity against H5N1 virus release significantly increases.

### Effect of anti-HA versus anti-NA antibodies in immune sera on virus release and entry

**Figure 1:**
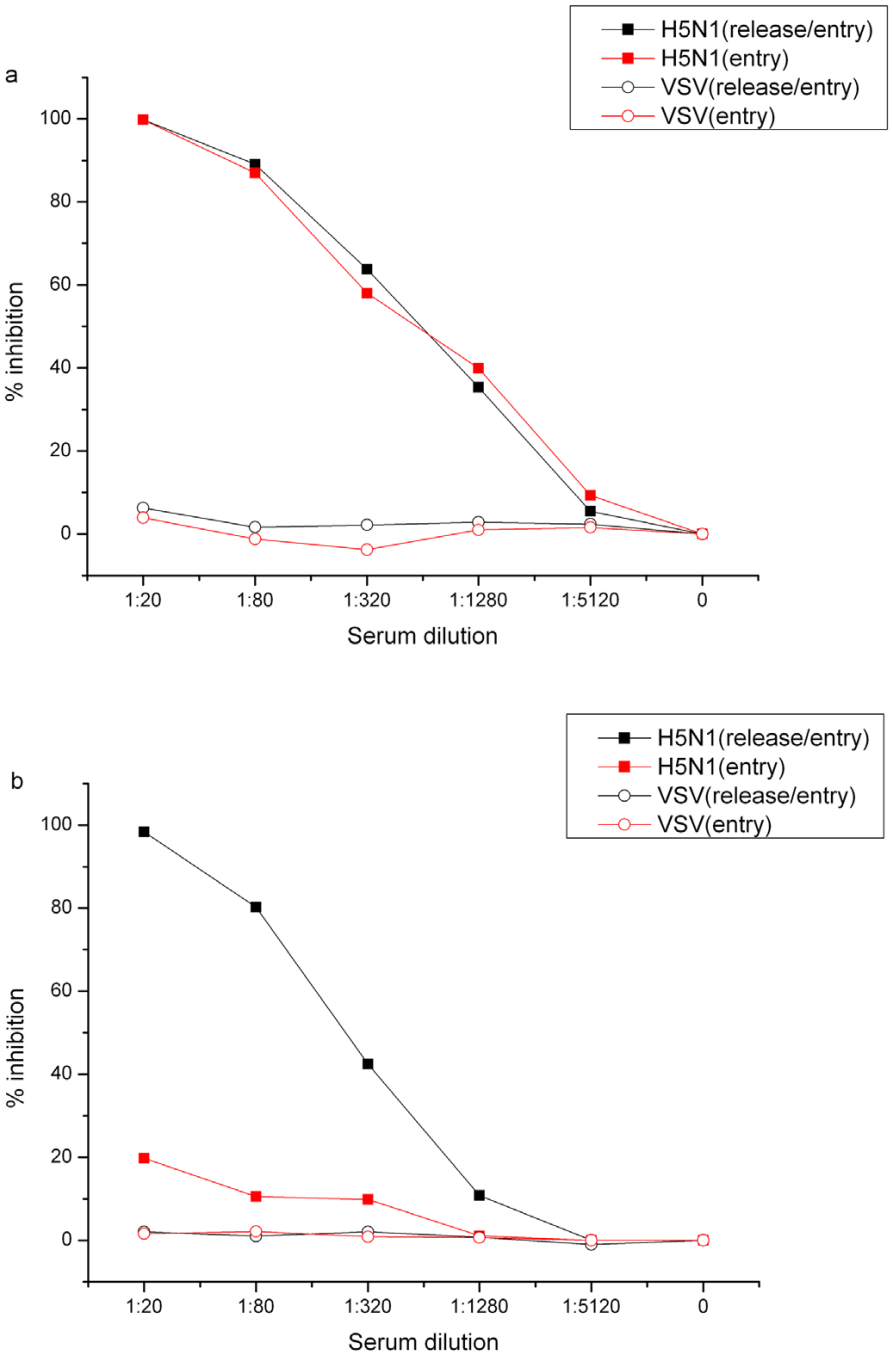
Dissect the role of anti-HA and anti-NA antibodies in influenza HA and NA pseudotype release and entry. a) Titration of anti-HA immune sera by PN entry and PN release/entry assays; b) Titration of anti-NA immune sera by PN entry and PN release/entry assays. Black close square stands for anti-H5N1 neutralizing antibody response measured by PN release/entry assay; Red close square stands for anti-H5N1 neutralizing antibody response measured by PN entry assay; Black open circle stands for anti-VSV-G neutralizing antibody response measured by PN release/entry assay; Red open circle stands for anti-VSV-G neutralizing antibody response measured by PN entry assay.

To better understand the contribution of anti-HA versus anti-NA antibodies in immune sera to virus release and to virus entry, we generated two immune sera against HA and NA, respectively, by immunizing female BALB/c mice with DNA plasmids expressing H5HA and N1NA, respectively. We then measured their neutralization activity by both PN entry and PN release/entry assays. Figure 1a shows that immune sera elicited with DNA plasmid expressing H5HA exhibited significant and similar neutralization activity when measured by both PN release/entry and PN entry assays. In contrast, immune sera elicited with DNA plasmid expressing N1NA exhibited significant neutralization activity when measured by PN release/entry assay; while measured by PN entry assay its neutralization activity was very limited (Figure 1b). Thus, these results indicate that anti-NA antibodies in immune sera mainly block virus release and have minimum effect on virus entry; whereas anti-HA antibodies in immune sera block virus entry, but not virus release. Thus, we conclude that the neutralization titers against H5N1 pseudotypes measured by PN entry assay (Table 2) truly reflected heterosubtypic anti-H5HA neutralizing antibody response; whereas the neutralization titers measured by PN release/entry assay (Table 4) reflected combined activity of both anti-NA neutralizing antibody response that blocks virus release and heterosubtypic anti-H5HA antibody response that blocks virus entry.

### Protection efficacy after the HPAI H5N1 challenge

Having demonstrated that low levels of heterosubtypic neutralizing antibody response against HPAI H5N1 are indeed elicited with seasonal influenza vaccine in humans, we next tested if such antibody response can also be elicited in mice and if so whether such antibody response offers any immune protection against lethal challenge of HPAI H5N1 viruses. To test this, we carried out two immunization and challenge experiments. In the first experiment female BALB/c mice randomly divided into three groups (5 mice per group) were injected i.m. twice with PBS, VLP expressing H5HA and N1NA or the seasonal influenza vaccine, respectively, followed by the challenge of 10 MLD_50_ HPAI H5N1 A/Shenzhen/406H/06 viruses. Dose 10 MLD_50_ was chosen to ensure 100% mortality rates in PBS control. In the second experiment 2 mice were injected i.m. twice with PBS, 2 mice with VLP and 22 mice with seasonal influenza vaccine followed by the same H5N1 challenge. Because both experiments showed similar trends of protection efficacy, we combined data of both experiments for analyses.

**Figure 2:**
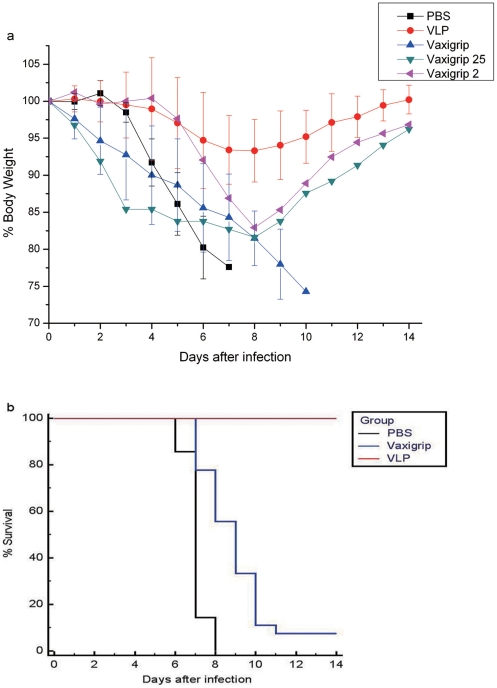
Protection efficacy elicited with H5N1 VLP and seasonal influenza vaccine in mice challenged with 10 MLD_50_ of HPAI H5N1 (A/Shenzhen/406H/06). a) Mean and standard deviation of percentage of original body weight in three groups as well as two individual mice (#2 and #25) after challenged with HPAI H5N1 virus. Black line: PBS control group; red line: VLP group; blue line: seasonal influenza vaccine group; purple line: mouse #2 vaccinated with seasonal influenza vaccine; green line: mouse #25 vaccinated with seasonal influenza vaccine. b) Survival rates after challenged with HPAI H5N1 virus. Survival rate was calculated based on percent survival within each experimental group. Black line: PBS control group; red line: VLP group; blue line: seasonal influenza vaccine group. The data shown here were the combined results of two individual experiments. The survival curves between different groups were compared by the log-rank test. *P* values between PBS and seasonal influenza vaccination groups, between PBS and VLP vaccination groups and between seasonal influenza and VLP vaccination groups were 0.0003, 0.0002 and <0.0001, respectively.

Figure 2a and 2b shows the time course of body weight change and the survival rate of each group during 14 days post challenge, respectively. In PBS control group, severe sickness of mice became evident, such as ruffling of the fur, anorexia and rapid weight loss, on day 4 after the challenge and between day 5 and 8 all 7 mice died. In VLP group, all 7 mice became mild sick on 7 to 9 days post challenge and mildly lost their body weight. After 10 days, all mice started gaining weight and survived. In seasonal influenza vaccine group, all 27 mice became sick and started loss of weight on day 4 or 6. Between day 7 and 9, 25 mice died. The remaining 2 mice (#2 and #25) regained their body weight on day 9 and survived.

### Neutralizing antibody responses and their protection outcome

**Figure 3:**
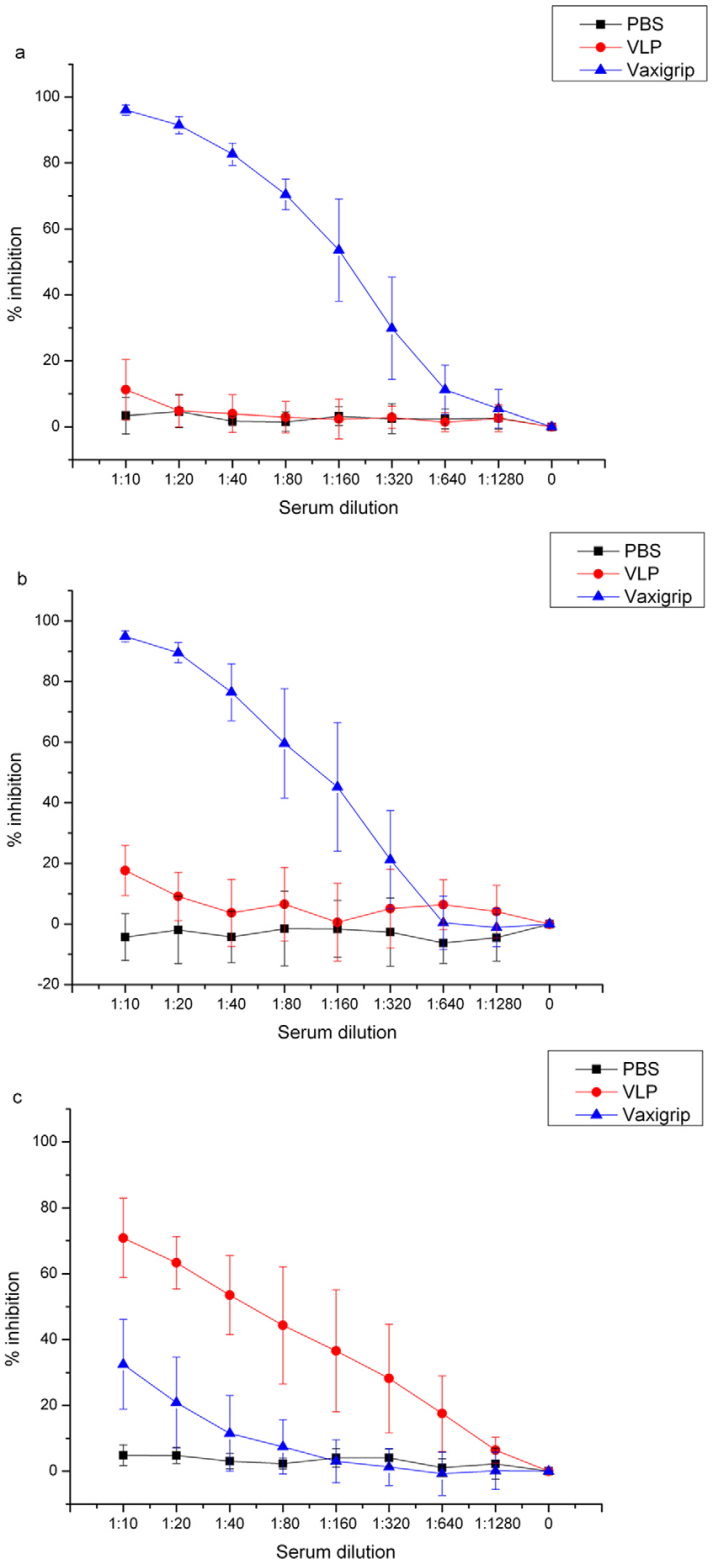
Mean and standard deviation of percentages of inhibition by mouse immune sera against influenza H1N1 (a), H3N2 (b) and H5N1 (c). PBS: sera from individual PBS control mouse group; VLP: immune sera from individual mice immunized with H5N1 VLP; Vaxigrip: immune sera from individual mice immunized with seasonal influenza vaccine. The data shown here were the combined results of two individual experiments.

To better understand the immune protection, we compared neutralizing antibody titers in post-boost immune sera in individual mice against H1N1 A/Brisbane/59/2007, H3N2 A/Brisbane/10/2007 and H5N1 A/Shenzhen/406H/06 pseudotypes using the PN entry assay. As expected, all mice received PBS did not have any neutralizing antibody activity against all three pseudotypes (Figure 3a, b and c). All mice primed and boosted with seasonal influenza vaccine exhibited high neutralizing antibody titers against H1N1 (Figure 3a) and H3N2 (Figure 3b), but relatively lower neutralizing antibody titers against H5N1 with exception of serum samples from mice #2 and #25 (Figure 3c). Interestingly, the mice #2 and #25 were the only two seasonal influenza vaccinated mice who survived from lethal challenge of HPAI H5N1 viruses (Figure 2). In contrast, all mice primed and boosted with VLP expressing H5HA and N1NA exhibited decent neutralizing antibody titers against H5N1 (Figure 3c), but none of them had measurable neutralization titers against H1N1 and H3N2 (Figure 3a and 3b).

**Figure 4:**
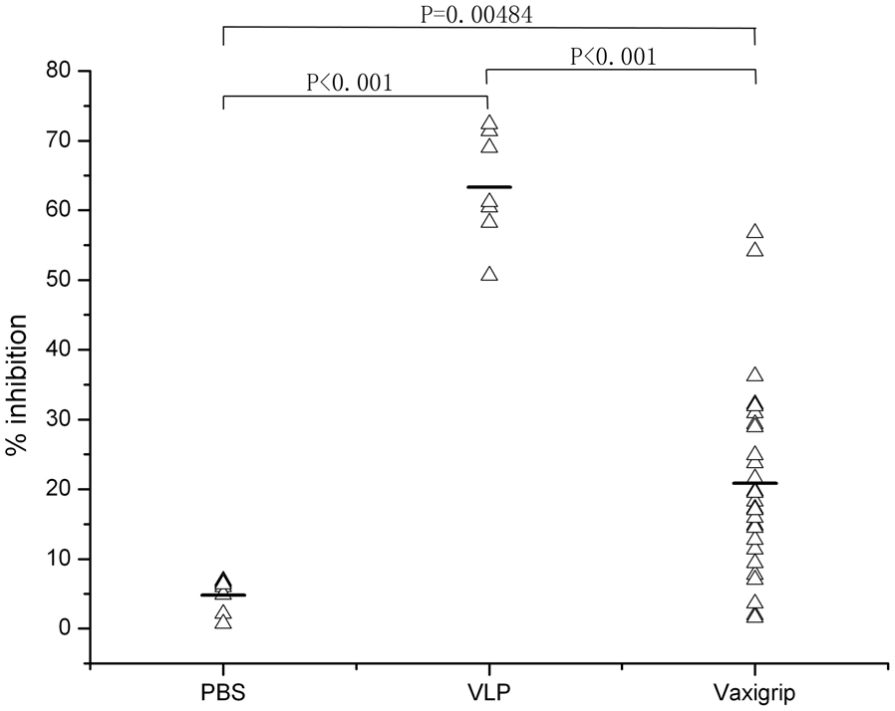
Statistical analysis of neutralizing antibody activity of immune sera at 1∶20 dilution among three groups. Horizontal bars show the mean values of percentage inhibition in each of three groups. The data shown here were the combined results of two individual experiments.

To further determine whether the levels of heterosubtypic neutralizing antibody responses elicited with seasonal influenza vaccine are statistically significant, we compared neutralization activity of immune sera among the three groups. Figure 4 shows neutralization activity at 1∶20 dilution of immune sera of individual mice among the three groups. Both Kolmogorov-Smirnov and Shapiro-Wilk normality tests show the normal distribution of neutralization activity among the three groups of serum samples (Heng *et al.* data not shown). Clearly, while significantly higher heterosubtypic neutralizing antibody responses were elicited with seasonal influenza vaccine as compared to PBS control (*P* = 0.00484), such antibody responses were also significantly lower than those elicited with homosubtypic H5N1 VLP (*P*<0.001). That may explain why 25 out of 27 mice after immunized with seasonal influenza vaccine still died after lethal H5N1 challenge. Interestingly, 2 (#2 and #25) out of 27 mice whose immune sera reached neutralization levels similar to H5N1 VLP controls survived from lethal H5N1 challenge. At 1∶20 dilution, sera from mice #2 and #25 exhibited 57% and 55% inhibition, respectively; while at the same dilution, immune sera from mice primed and boosted with H5N1 VLP exhibited 50 to 72% inhibition.

## Discussion

Heterosubtypic neutralizing antibody response between the annual seasonal influenza vaccine and other potential pandemic influenza viruses such as HPAI H5N1 viruses has important implications for public health pandemic influenza preparedness. In the present study, we demonstrated that in most human individuals 2008–2009 seasonal influenza vaccination elicited low, but measurable, heterosubtypic neutralization activity against various clades and subclades of H5HA and this heterosubtypic neutralization activity can be detected by a sensitive PN assay, but not by MN assay. Thus, on one hand our results (Tables 1, 2 and 4) agree with what was reported by Gioia *et al* [6], Corti *et al* [8], Garcia *et al* [15] and Kositanont *et al* [16] that indeed there is heterosubtypic neutralizing antibody response between the seasonal influenza vaccine with HPAI H5N1 viruses. On the other hand, our results (Table 3) also agree with what was reported by Tang *et al* [7] that the neutralization activity was not detected by MN assay.

The PN assay is not only more sensitive than conventional MN and HI assays, but also allows us to dissect whether the neutralizing antibody responses act on virus release or virus entry or both (see Materials and Methods for detail assay description). In the present study, by measuring pre- and post-human immune sera as well as mouse immune sera against N1NA or H5HA with both PN entry and PN release/entry assays (Tables 1, 2 and 4 and Figure 1) we were able to conclude that seasonal influenza vaccination elicits both homosubtypic anti-N1NA and heterosubtypic anti-H5HA antibodies in human immune sera. The former blocks virus release; whereas the latter blocks virus entry.

A number of studies in animals have shown that cross-protective immunity against H5N1 can be elicited with seasonal influenza vaccines or infection [17]–[25]. Although majority of such cross-subtypic protection are thought to be mediated by T cells against shared conserved epitopes in viral internal proteins such as NP and M1 between seasonal influenza vaccine and H5N1 viruses [26]–[31], several studies did show seasonal influenza vaccination elicited B cell responses [17], [19], [23], [24], [25]. For example, one study showed that priming animals with trivalent live attenuated seasonal influenza vaccine indeed significantly increased anti-H5N1 B cell responses [19]. Another studies using gene-targeted B cell (igH-6^−/−^) or β2-microglobulin (β2m^−/−^) deficient mice showed that heterosubtypic immune protection against lethal challenge of HPAI H5N1 viruses elicited with intranasal immunization of inactivated influenza H3N2 vaccine plus LT (R192G) adjuvant was mediated by B cells, but not by CD8^+^ T cells [25]. Moreover, antibody response induced by the influenza A virus N1NA affords partial protection against H5N1 viruses in mice and such antibody response was detected in humans with no history of H5N1 exposure [17], [23]. Finally, parental immunization of CD1 mice with trivalent seasonal influenza vaccine elicited heterosubtypic H5-reactive antibodies that confer partial protection against H5N1 influenza challenge [24].

Although these aforementioned studies strongly suggested that antibody responses elicited with seasonal influenza vaccines confer certain degree of protection against heterosubtypic H5N1 challenge, what neutralizing antibody titers were required for such protection was not clear. In the present study, the sensitive PN assay enables us to detect low levels of heterosubtypic neutralizing antibody responses in mice elicited with seasonal influenza vaccine (Figure 3c). Among them 2 mice whose immune sera at 1∶20 dilution exhibited higher than 50% heterosubtypic neutralizing antibody activity actually survived from lethal challenge of H5N1 virus; while all remaining 25 mice whose heterosubtypic neutralizing antibody activity at 1∶20 dilution was lower than 50% inhibition died (Figures 2b and 4). Actually, the heterosubtypic neutralizing antibody activity against H5N1 detected in these two survived mice were similar to that detected in H5N1 VLP vaccinated mice (Figure 3b). Taken together, these results did indicate that when heterosubtypic neutralizing antibody activity in immune sera at 1∶20 dilution is equal to and higher than 50% inhibition, such levels of neutralizing antibody activity may be indeed high enough to offer protection against severity of diseases caused by lethal challenge of highly pathogenic H5N1 virus.

Finally, in the present study we also showed that in most human cases immune sera elicited with seasonal influenza vaccination had several fold higher heterosubtypic neutralizing antibody responses against H5N1 than those found in two survived mice mentioned above (see Table 2). Therefore, if the levels of heterosubtypic neutralizing antibody activity that protect mice from lethal challenge of H5N1 virus could be extrapolated in humans, seasonal influenza vaccination may also offer immune protection against severity of HPAI H5N1 infection in humans.
